# The Effect of Cross-Level Interaction between Community Factors and Social Capital among Individuals on Physical Activity: Considering Gender Difference

**DOI:** 10.3390/ijerph16030495

**Published:** 2019-02-11

**Authors:** Hee-Jung Jun, Seoyeon Park

**Affiliations:** Department of Public Administration and Graduate School of Governance, Sungkyunkwan University, Seoul 03063, Korea; suyon1541@gmail.com

**Keywords:** physical activity, social capital, community factor, cross-level interaction, gender difference

## Abstract

This study examines the effect of cross-level interaction between community physical environment and social capital among individuals on physical activity by considering gender difference. In this regard, we ask two research questions: (1) What is the effect of cross-level interaction between community factors and social capital among individuals on physical activity? (2) Is there gender difference in the effect of the cross-level interaction? To examine the research questions, this study used the 2015 Korea Community Health Survey and used multi-level analyses. The empirical analyses show that while there are both positive and negative cross-level interaction effects between physical activity-supportive community environment and social capital among individuals on physical activity, the positive cross-level interaction effect is more pronounced for women than for men. These findings suggest that local efforts to improve public health should take into account the cross-level interaction effect between community physical environment and social capital among individuals as well as gender difference.

## 1. Introduction

Physical inactivity can be a fundamental cause of various public health problems such as obesity, diabetes, high blood pressure, and even cancer [1,2,3], therefore, a large number of studies have examined why some people are physically active and others are not. In this study, we focus on the effect of cross-level interaction between community physical environment and social capital among individuals on physical activity and examine gender difference in the cross-level interaction effect. In this regard, we ask two research questions: (1) What is the effect of cross-level interaction between community factors and social capital among individuals on physical activity? (2) Is there gender difference in the effect of the cross-level interaction? 

Earlier studies on physical activity have focused on individual-level factors. Studies find that demographic characteristics such as age, gender, race/ethnicity, and marital status are related to physical activity [4,5,6,7]. In addition, there are studies which find that higher socioeconomic status (SES) leads to higher physical activity because affluent people have more resources to engage in physical activity and highly educated people are more likely to incorporate healthy behaviors into their lifestyle [8]. 

Recent studies examine the role of social capital on various health outcomes and find that a greater level of social capital leads to favorable health outcomes [9], such as reduction in mental health illnesses [10,11,12], risk of heart disease [13], and risk of obesity [14,15]. Social capital refers to social connections and relations among individuals and groups and its major components include social network, trust, and norms of reciprocity [16,17,18]. Social capital can be formed at the both individual and community levels [19] and plays a role as all kinds of resources to produce socio-economically and politically desirable outcomes [20,21]. With regard to physical activity, studies find that social trust is positively associated with walking and other sports-related activities, which reduces the risk of physical inactivity [22,23]. Legh-Jones and Moore’s study [24] emphasized the importance of network social capital in addition to social participation on physical activity. In addition, Lindstrom et al. [25] found that social participation can remove socioeconomic status differences in leisure-time physical activity, although people with low socio-economic status are often associated with a low degree of leisure time activity. These findings suggest that social capital can be an alternative that can increase physical activity among people with low socio-economic status. 

Then, what is the mechanism by which social capital positively affects the degree of physical activity? According to Kawachi and Berkman [26], healthy behaviors such as physical activity and reducing caloric intake are more likely to be promoted among people who are socially connected. Drawing from the diffusion of the innovation theory, Rogers [27] argues that there is more rapid diffusion about health-related information among people who are socially connected compared to those who are socially isolated. In addition, informal social control prevents deviant health-related behaviors, such as no physical activity and high caloric intake [26]. Psychological processes among socially connected people that provide mutual support to each other can also lead to positive changes, such as reduced caloric intake and increased physical activity [26]. 

On the other hand, a group of studies focus on the influences of community-level factors on health outcomes. More specifically, those studies have found that the community’s physical environment characteristics, such as availability of parks and recreational facilities, pedestrian-friendly environment, and land-use mix promote physical activity such as walking and exercising [4,28,29,30,31,32,33,34]. In addition to community physical environment, studies have also found that community socio-economic environment can affect the degree of individual physical activity. For example, Adler [35] finds that social inequalities such as unequal distribution of income can adversely influence health with increased exposure to stressful events and reduced access to health facilities. 

One area that has not been sufficiently explored is if there is a cross-level interaction effect between community-level physical environmental factors and social capital among individuals. For example, social capital among individuals may promote physical activity further by encouraging interactions with community physical environment that also promote physical activity. Finding a positive cross-level interaction effect between physical activity-supportive community environment and social capital among individuals on physical activity may answer the question as to when the best outcome toward increased physical activity can be achieved. There are studies analyzing the influences of both individual-level and community level-social capital on public health. In this study, we examine the effect of individual-level social capital on physical activity as we consider gender difference, which is an individual-level factor. Based on the logic of the discussion, we propose our 1st hypothesis that:

**H1.**  *There is a positive effect of cross-level interaction between physical activity-supportive community environment and social capital among individuals on physical activity.*

On the other hand, the cross-level interaction effect between community physical environment and social capital among individuals may differ by gender. In general, compared to men, women report a higher level of social capital [36]. More specifically, social capital within a community is likely higher for women than men. This is most likely due to women spending more time in their localities. Compared to men, women are less likely to work, and social roles, such as childrearing and maintaining the household are usually fulfilled by the women [31,37]. Additionally, a number of studies report gender differences in the effect of community factors on health outcomes. For example, Molinari et al. [38] find that social quality of local environment is more important for perceived health in women while physical quality of local environment is more important for perceived health in men. Ellaway and Macintyre [39] find that there is a stronger association between perceptions of neighborhood social cohesion and mental health for men than for women while there is a stronger association between local neighborhood problems (e.g., lack of facilities, physical features) and physical health for women than for men. In supporting the position that women’s health is more associated with community characteristics than men’s health, more studies find a stronger negative association between deprived neighborhood features and good health for women than for men (e.g., [31,36,37]). Given that social capital within a community is likely higher among women than men, the stronger relationship between community characteristics and women’s health suggest that there is a greater positive effect of cross-level interaction between physical activity-supportive community environment and social capital among individuals in the women’s group than in the men’s group. Based on this logic, we propose our 2nd hypothesis that:

**H2.**  *The positive effect of cross-level interaction between physical activity-supportive community environment and social capital among individuals on physical activity is greater for women than for men.*

## 2. Materials and Methods

### 2.1. Materials

This study aims to examine the effect of cross-level interaction between community factors and social capital among individuals on physical activity and gender difference in the effect of cross-level interaction. For the empirical analysis, we used the 2015 Korea Community Health Survey (KCHS) that was conducted by the Korea Centers for Disease Control and Prevention as the principal dataset. The KCHS is a nationwide survey that targets adults aged 19 years and older, and is an annual survey that has been taken since 2008. Although the latest survey was taken in 2016, we used the 2015 survey data for the empirical analyses. It was because the 2016 survey did not ask about social capital and using the 2015 KCHS data are more comparable to the community-level data that were mostly taken in 2015. In addition to social capital questions, the survey asked about physical activity, health status questions regarding obesity, mental health, and general health, as well as demographic and socio-economic characteristics. 

The sample size of the KCHS data is 228,558, which includes 102,829 men and 125,729 women. To be a nationwide representative sample, the survey was taken via the following multi-stage sampling process: at first, sub-units within the areas where each community health center covers were randomly selected; in the second stage, each sub-unit was divided into more specified areas, including areas for high-rise housing complexes and general residential areas; and at the final stage, households were randomly selected from areas for high-rise housing complexes and general residential areas based on the official registry of residents.

The dependent variable in this study is individual physical activity and a dichotomized variable, either high/moderate physical activity or low physical activity/inactivity. To assess individual physical activity level, we used the International Physical Activity Questionnaire (IPAQ) scoring system that was also incorporated by the KCHS in its survey and used in other studies (e.g., [24,40]). Then, we considered the types of physical activity defined by the IPAQ, which includes walking, moderate, and vigorous activities: walking activities include walking for transport, exercising, and commuting; moderate activities are defined activities that make breathing somewhat harder than normal such as slow swimming, doubles tennis, volley ball, and table tennis; and vigorous activities are defined activities that make breathing much harder than normal such as running, fast cycling, fast swimming, soccer, basketball, singles tennis, squash, and jump-ropes. 

Then, each activity type was converted to the metabolic equivalent of task (MET): walking activity is equivalent to 3.3 MET; moderate activity is equivalent to 4 MET, and vigorous activity is equivalent to 8 MET. Finally, following IPAQ Research Committee [41], we categorized each respondent into high, moderate, and low physical activity, using the calculated MET min/week): high level of physical activity was defined as at least 3 days of vigorous activity achieving at least 1500 MET min/week, or 7 or more days of any combination of walking, moderate or vigorous activities accumulating at least 3000 MET min/week; moderate level of physical activity was defined as 3 or more days of vigorous activity for at least 20 minutes per day, 5 or more days of moderate activity and/or walking for at least 30 minutes per day, or 5 or more days of any combination of walking, moderate or vigorous activities achieving at least 600 MET min/week; and low level of physical activity was defined as activity that do not belong to any of moderate or high level of physical activity. 

The key independent variables in this study are the components of social capital. As defined by Putnam [18], we used the levels of trust, reciprocity, and network in one’s community that each respondent assesses as the social capital variables in our models. Trust was measured by the item “people in my neighborhood can be trusted” (yes = 1/no = 0). Reciprocity was measured by the item “people in my neighborhood help each other when they have family events” (yes = 1/no = 0). Network was assessed by the question “how often do you contact your neighbors?” and this question was measured as an ordinal variable, which ranges from 1 to 6 (never = 1, once a month = 2, 2–3 times a month = 3, once a week = 4, 2–3 times a week = 5, and 4 or more times a week = 6). 

We also included demographic and socio-economic characteristics at the individual level as control variables as previous studies find that those variables determine individual physical activity [4,5,6,7]. Those control variables include age, age squared, gender (men = 1/women = 0), educational level (i.e., length of year educated), working status (working = 1/not working = 0), length of residence (in the number of year), job type (manual job = 1/non-manual job = 0), household income (household monthly income ranged from less than 500 (1), 500–1000, 1000–2000, 2000–3000, 3000–4000, 4000–5000, 5000–6000, and over 6000 (8) US dollars), and household size. Finally, we controlled for subjective health status (healthy = 1/not healthy = 0) as it can also affect physical activity. 

Data at the community level were collected from the Korean Statistical Information Service (KOSIS) and Local Finance Integrated Open System. While there is a total of 254 health centers, which translates to 254 communities in our study, we excluded 29 communities from the total. It was because either the excluded communities were islands, which can result in atypical patterns, or the data were simply not available for those communities. As a result, from the total of 254 communities, we used 225 communities for the empirical analyses. We included population density (logged), level of land-use mix, the area (m^2^) of park per person, and the number of sports facilities per 1000 people as community physical environment factors. The level of land-use mix was computed using Bhat and Guo’s method [42]:

Li=1−{|rL−13|+|mL−13|+|oL−13|43}

where *L_i_* is the mixed land use index, *L* is the total land size, *r* is the size in residential land use, *m* is the size in commercial/industrial land use, and *o* is the size in other land-uses. According to the equation, this variable ranges between 0 and 1, and 0 indicates perfect homogeneity and 1 indicates perfect diversity in land use. 

In Korea, high-rise housing complexes are more preferred compared to general residential areas, because it provides the living environment that promotes more physical activity in sidewalks, gyms, and other recreational facilities [43]. Thus, we included percentage of high-rise housing complex area to the total residential area. Additionally, to control for socio-economic status of a community, we included the fiscal self-reliance ratio, which is the ratio of communities’ own revenue sources such as local taxes to the total revenue. The fiscal self-reliance ratio indicates socio-economic status as well as the level of fiscal strength of the communities. Table 1 shows the descriptive statistics of the variables included in this study for the pooled, men, and women models. We also ran the t-test for continuous variables and *x*^2^ test for categorical variables to compare characteristics between men and women at the individual level. 

As shown in Table 1, at the individual level, men and women’s groups are statistically significantly different from each other. First, there is a greater share of people who are physically active for men than women. This is consistent with Lee et al. [40] finding that there is a higher share of people who meet physical activity at the recommended level for men than for women. Regarding social capital variables, as women in many countries stay in their localities for a longer period [31,36], women are more likely to have trust and reciprocity among individuals in their neighborhoods, and consequently, the level of network within the neighborhood is greater for women than for men. Regarding other individual variables, educational level and shares of people working and having a manual job are greater for men than women, which also resembles many other countries [44]. Household income is greater in the men’s group than the women’s group, which is consistent with lower income given to women in many countries. Household size is greater in the men’s group than in the women’s group, which may be due to women living longer than men, and thus men having more of a likelihood to live with the other family members. Finally, there is a greater share of people in the men’s group than the women’s group who indicated they are healthy. 

### 2.2. Methods

To test the proposed hypotheses, we employed multi-level modeling as the data and hypotheses have a hierarchical structure. Running a standard ordinary least squares (OLS) model after disaggregating and attaching community-level data to the individual level introduces the possibility of biased standard errors because observations within each community is not independent. However, a multi-level model handles hierarchically structured data better because it can minimize the above mentioned statistical issue in running an OLS regression model. In this study, as the dependent variable is a dichotomous variable (i.e., being physically active or not), we ran a multi-level logit analysis.

To test the first hypothesis that *there is a positive effect of cross-level interaction between physical activity-supportive community environment and social capital among individuals on physical activity*, we ran level-2 random intercept models, which allow intercepts to vary across communities. Individual-level and community-level factors are level-1 and level-2 variables in the multi-level models, respectively. As this study hypothesizes that there is a cross-level interaction effect between community factors and social capital among individuals on physical activity, we also included cross-level interaction terms which interact physical activity-supportive community environment, including population density, land-use mix, the area of parks, the number of sports facilities, and percentage area for housing complexes at the community level with separate models for trust, reciprocity, and network at the individual level. We set separate models for trust, reciprocity, and network variable rather than a model that included all three of the variables. This was because including all of the social capital components and their cross-level interaction terms with community factors into one model could make the model very complex and may cause a colinearity problem.

To test the second hypothesis that *the positive effect of cross-level interaction between physical activity-supportive community environment and social capital among individuals on physical activity is greater for women than for men*, we ran separate multi-level analyses between men and women in addition to the pooled model. By running separate analyses, we wanted to examine gender difference in the cross-level interaction effect between community physical environment and social capital among individuals on physical activity. In the next section, we discuss the results of the empirical analysis in the order of trust, reciprocity, and network.

## 3. Results

### 3.1. ANOVA Model

In a multi-level analysis, it is conventional to check if a significant portion of variation in the dependent variable is attributed to the larger-context differences. Therefore, we first ran an unconditional model without any explanatory variables, which is a one-way ANOVA model with random effects. According to the variance components, 6.1% of total variation in the pooled model, 6.1% in the men model, and 6.5% in the women model are attributed to community-level differences (In a multilevel logit model, the variance at level-1 for a binary outcome is normalized to equal π^2^/3 or 3.29). Although the community-level variances are not very high, they are statistically significant, which means that there are statistically significant variations at the community level across the models. In addition, it is normal that a larger share of variation in the dependent variable is attributed to the individual-level differences as the dependent variable was measured at the individual level. Table 2 shows the results of the ANOVA models. 

### 3.2. Trust Model

Table 3 shows multi-level estimates for the trust model. At level 1, trust, a key independent variable, is statistically significant and positively related to physical activity in all of the pooled, men, and women models. Individuals who have trust in the community are more likely to engage in physical activity. At level 2, population density and the number of sports facilities are negatively related to physical activity in the women’s model but are not statistically significant in both of the pooled and men models. While higher population density may indicate a more walking-friendly environment by having more sidewalks and providing easier access to public transits in countries like the United States, that does not seem to be the case in Korea. Given that Korea is one the top countries in terms of yearly working hours among OECD countries [45], high population density in Korea may indicate a busy lifestyle for the workers who do not have enough time for physical activity. Additionally, the number of sports facilities is negatively related to physical activity. This suggest that a large number of sports facilities do not necessarily increase individual physical activity. This may also indicate that the quality of the sports facilities actually matters more than the quantity of these facilities in increasing individual physical activity. Land-use mix in the pooled and women models and percentage area for housing complexes in all models are positively related to individual physical activity. 

Regarding the cross-level interaction variables, while the number of sports facilities with individual-level trust was the only statistically significant variable for men, more cross-level interaction variables were statistically significant for women. In the women model, the cross-level interaction variables of the area of parks, the number of sports facilities, and percentage area for housing complexes with individual-level trust are positively related to physical activity. Given the area of parks itself was not statistically significant in the women model, the positive cross-level interaction terms between the area of parks with individual-level trust implies that the increase in the area of parks alone do not promote women’s physical activity, but can increase their physical activity when there is trust between the community members. While the cross-level interaction variable between the number of sports facilities and individual-level trust is negatively related to physical activity in both of the pooled and men models, the same variable is positively related to physical activity in the women model. The positive cross-level interaction term between the number of sports facilities and individual-level trust in the women model mediate the negative effect of the number of sports facilities in the women model. Finally, the cross-level interaction effect between percentage area for housing complexes and individual-level trust had a positive interaction only in the women model. Given that percentage area for housing complexes is positively related to individual physical activity in the women model, the cross-level interaction with individual-level trust further enhances physical activity for women. In other words, those who live in an area with a large share of housing complexes are more likely to engage in physical activity and their physical activity is further enhanced when having trust in community members. However, this can be a unique pattern that may not be found in countries such as the United States, where high-rise housing complexes are less preferred compared to detached single-family housing units. 

While the effects of some individual-level variables are not very different between men and women, other variables such as, age, age squared, and length of residence show differences between men and women. While age is negatively and positively related to men and women, respectively, age squared term is positively and negative related to men and women, respectively. In other words, men and women show different patterns in physical activity as they get older. In addition, length of residence was statistically significant with a positive relationship only in the pooled and women models. This may be associated with the fact that women stay longer in their communities than men do [24] and thus have more attachment to the community over time, which can positively affect physical activity. For the reciprocity and network models, the results are very similar to the trust model, except for the working status variable that is statistically significant with a negative relationship only for the men model in the network model while the same variable is negatively related to physical activity for both of the men and women models in the trust and reciprocity models. 

Our first hypothesis, *there is a positive effect of cross-level interaction between physical activity-supportive community environment and social capital among individuals on physical activity*, was supported by the cross-level interaction of the area of parks, the number of sports facilities, and percentage area for housing complexes with trust in the women model. As the positive effect of cross-level interaction between physical activity-supportive community environment and individual-level trust is statistically significant mostly for women, these results support our second hypothesis, *the positive effect of cross-level interaction between physical activity-supportive community environment and social capital among individuals on physical activity is greater for women than for men*.

### 3.3. Reciprocity Model

Table 4 shows the multi-level estimates of the reciprocity model. At level 1, reciprocity is statistically significant with a positive sign in all of the pooled, men, and women models. In other words, those who are socially connected through reciprocity in their communities are more likely to engage in physical activity, and this result is consistent with the trust model. At level 2, population density is negatively related to individual physical activity in all of the pooled, men, and women models and the results are consistent with the effect of population density in the trust model for women. Land-use mix and fiscal self-reliance ratio are positively related to individual physical activity in the pooled and women models but not in the men model. Percentage area for housing complexes is positively associated with physical activity in all of the pooled, men, and women models.

Regarding the cross-level interaction variables, although the area of parks at the community level was not statistically significant, its interaction term with reciprocity at the individual level was statistically significant with a positive sign in the pooled and women models. This result implies that a larger area for parks does not simply promote physical activity, which is consistent with what was found in the trust model. People are more likely to engage in physical activity when both a larger area for parks and social capital among individuals are available within a community. Other cross-level interaction variables show different patterns from the trust model. The cross-level interaction term between population density and individual-level reciprocity was statistically significant with a positive sign only in the pooled and men models, but not in the women model, which implies that there is a mediating effect given that population density itself is negatively related to physical activity. Unlike in the trust model, where the number of sport facilities variable was not statistically significant, the cross-level interaction term between the number of sports facilities and individual-level reciprocity was negatively related to physical activity in all of the pooled, men, and women models. In other words, this negative relationship may indicate that the quantity of sport facilities does not simply promote physical activity but rather indicates a presence of more complex dynamics. While the cross-level interaction variable between percentage area for housing complexes and individual-level reciprocity was not statistically significant in the women model, it was negatively related with physical activity in the pooled and men models. Given that percentage area for housing complexes was positively related to physical activity in all of the pooled, men, and women models, this finding indicates that the positive effect of percentage area for housing complex is maintained only in women. 

The reciprocity model is relatively weaker than the trust model in supporting the proposed hypotheses. The first hypothesis that *there is a positive effect of cross-level interaction between physical activity-supportive community environment and social capital among individuals on physical activity* was only supported by the positive cross-level interaction variables of community-level population density in the pooled and men models and area of parks in the pooled and women models with individual-level reciprocity. In addition, the second hypothesis that *the positive effect of cross-level interaction between physical activity-supportive community environment and social capital among individuals on physical activity is greater for women than for men* was only supported by the positive cross-level interaction term between the area of parks and individual-level reciprocity in the women model, which is consistent with the trust model for women. 

### 3.4. Network Model

Table 5 shows the multi-level estimates of the network model. At level 1, network is statistically positively related to physical activity in all of the pooled, men, and women models. This result suggests that as individuals have increasing networks with their neighbors, the more likely these individuals will engage in physical activity. This result is also consistent with the results, which were shown in the previous sections that individual-level trust and reciprocity are positively related to physical activity. At level 2, population density in the pooled and women models are negatively related to individual physical activity, which is similar to the trust and reciprocity models. In addition, the number of sports facilities are negatively related to physical activity in both of the pooled and women models as in the trust model for women. Similar to the trust and reciprocity models, land-use mix and percentage housing complexes are positively related to physical activity in all of the pooled, men, and women models. 

Regarding the cross-level interaction, the interaction term between the area of parks and individual-level network showed a consistent result as in the trust and reciprocity models. In other words, while the area of parks itself is not statistically significant, its cross-level interaction with individual-level network is statistically significant with a positive sign. Thus, a larger area of parks does not necessarily promote physical activity for women. However, when there exists both a larger area of parks and a high network level in the community, women are more likely to engage in physical activity. The cross-level interaction term between the number of sports facilities and individual-level network is not statistically significant in both of the pooled and women models, but statistically significant in the men model with a negative sign, which is similar to the trust and reciprocity models. 

The network model reports similar results to those given in the trust and reciprocity models. We found that the availability of parks does not directly promote physical activity. Rather, parks are useful in promoting physical activity when combined with social capital. This finding supports our first hypothesis that *there is a positive effect of cross-level interaction between physical activity-supportive community environment and social capital among individuals on physical activity*. In addition, the fact that a positive cross-level interaction effect was found only in the women model (i.e., between the area of parks and individual-level network) supports the second hypothesis that *the positive effect of cross-level interaction between physical activity-supportive community environment and social capital among individuals on physical activity is greater for women than for men*.

## 4. Discussion

Physical activity is important as it can prevent various health problems. Numerous studies examine factors affecting physical activity and find that both community physical environment and social capital among individuals influence physical activity. Despite numerous studies, few studies examine the effect of cross-level interaction between community physical environment and social capital among individuals on physical activity. Our study aims to fill the gap in the literature by examining not only the cross-level interaction effect between physical activity-supportive community environment and social capital among individuals but also its gender difference. 

Our study supports previous studies’ findings which state the importance of social capital and community physical environment on physical activity. We found that all of trust, reciprocity, and network among individuals, the components of social capital, were positively related to physical activity. In other words, individuals who have a higher level of trust, reciprocity, and network with neighbors are more likely to engage in physical activity. We also confirmed that a considerable share of variance in physical activity is attributed to community-level differences. 

The first hypothesis that *there is a positive effect of cross-level interaction between physical activity-supportive community environment and social capital among individuals on physical activity* was supported by the positive cross-level interaction effect between the area of parks and social capital in all of the trust, reciprocity, and network models for women. In addition, there was a positive cross-level interaction effect between population density and reciprocity among individuals for pooled and men and between the number of sports facilities and trust among individuals for women. Each of the area of parks, population density, and the number of sports facilities variables at the community level were either not statistically significant or negatively related to physical activity. Therefore, the positive cross-level interaction effects suggest that parks, population density, and sports facilities themselves do not directly promote physical activity but rather they can either promote physical activity or lower the negative effect of the community factors when combined with social capital. In addition, the positive effect of percentage area for housing complexes is further enhanced by cross-level interacting with trust for women given that percentage area for housing complexes itself is positively related to physical activity in the same model. Overall, these results suggest that localities will need to take into account both community’s physical environment and social capital among individuals to successfully promote physical activity. 

The empirical analyses also report that more cross-level interaction variables are statistically positively related to physical activity across the trust, reciprocity, and network models for women than for men. In particular, the cross-level interaction variables between the area of parks and trust, reciprocity, and network among individuals were statistically significant with a positive sign only in the women models, not in the men models. These results suggest that there is gender difference and support the second hypothesis that *the positive effect of cross-level interaction between physical activity-supportive community environment and social capital among individuals on physical activity is greater for women than for men.* Given these findings that there are gender differences in the cross-level interaction effect on physical activity, we suggest that gender differences should be considered in local efforts to promote physical activity. 

Although this study aims to fill the gap in the physical activity literature, there are still some limitations. The KCHS data, the principle data in this study, provides the necessary information that allows examining the association between social capital and physical activity. However, the dataset does not provide the information where individual respondents are located at the neighborhood level, but instead only at the lower-level local government, which can be much broader than a neighborhood. As individuals often engage in physical activity within a neighborhood, the variability in physical activity-supportive environment across neighborhoods may need to be taken into account in examining the association between community characteristics and individual physical activity. In addition, we used population density as a proxy of community walkability as denser communities are often characterized with more sidewalks and greater availability of public transportation. As population density was negatively related to individual physical activity, it seems that future studies will need to employ better measure for walkability. In addition, future studies will need to differentiate the types of parks and sports facilities, as different types of parks and sports facilities can influence physical activity differently. Finally, while this study found that social capital is a major factor for increasing physical activity, this study did not test the alternative relationship that physical activity can lead to higher social capital. As Leyden [46] and Jun and Hur [47] find, social capital can be enhanced by walking, a kind of physical activity. For more comprehensive understanding of the relationship between community factors, social capital, and physical activity, future studies will need to test the alternative relationship.

## 5. Conclusions

In this study, we asked two research questions, (1) What is the effect of cross-level interaction between community factors and social capital among individuals on physical activity? (2) Is there gender difference in the effect of the cross-level interaction? To examine the research questions, we analyzed the 2015 Korea Community Health Survey data and used multi-level analyses. The empirical analyses show that while there are both positive and negative cross-level interaction effects between community factors and social capital among individuals on physical activity, the positive cross-level interaction effect is more pronounced for women than for men, thereby showing gender difference in physical activity. These results suggest that public health policies should reflect the cross-level interaction effect between community physical environment and social capital among individuals as well as gender difference. In other words, the promotion of individual physical activity can be better achieved when both physical activity-supportive community environment is equipped and social capital among individuals is higher. Also, policy makers should acknowledge that this kind of interaction between physical activity-supportive community environment and social capital among individuals is more effective in increasing individual physical activity for women than for men. The findings in this study may not be directly applicable to other countries. Nevertheless, this study will provide useful insights for other countries, especially developing Asian countries, in setting public health policies that can get more people engaged in physical activity.

## Figures and Tables

**Table 1:** Descriptive statistics.

	Variables	Pooled		Men		Women		t-/*x*^2^ Test
		Mean	S.D.	Mean	S.D.	Mean	S.D.	
Individual Level Level 1	Physical activity (high/moderate = 1)	0.58	-	0.62	-	0.54	-	1384.75 ***
	Trust (yes = 1)	0.69	-	0.68	-	0.71	-	202.46 ***
	Reciprocity (yes = 1)	0.55	-	0.54	-	0.56	-	48.24 ***
	Network	3.80	2.13	3.50	2.15	4.03	2.07	−59.01 ***
	Age	53.15	17.26	52.14	16.82	53.96	17.56	−24.76 ***
	Gender (men = 1)	0.45	-	-	-	-	-	
	Education	10.86	4.70	11.98	3.95	9.96	5.05	103.99 ***
	Working status (working = 1)	0.63	-	0.76	-	0.52	-	13,541.12 ***
	Length of residence	3.97	1.50	3.98	1.50	3.96	1.49	1.77 *
	Job (manual = 1)	0.31	-	0.44	-	0.22	-	12,341.15 ***
	Household income	4.21	2.02	4.36	1.95	4.10	2.06	30.28 ***
	Household size	2.86	1.31	2.91	1.27	2.81	1.35	16.81 ***
	Subjective health (healthy = 1)	0.78	-	0.83	-	0.74	-	2245.28 ***
Community Level Level 2	Population density (logged)	3.85	7.67					
	Land-use mix	0.33	0.16					
	Area of parks (per person)	21.33	22.49					
	Number of sports facilities (per 1000 people)	2.07	6.25					
	% area for housing complexes	0.18	0.14					
	Fiscal self-reliance ratio	0.26	0.14					
	Number of observations	Level 2: 225		Level 2: 225		Level 2: 225		
		Level 1: 202,371		Level 1: 90,623		Level 1: 11,1748		

*** *p* < 0.01, * *p* < 0.1.

**Table 2:** ANOVA Models.

**Pooled model**				
Fixed effect	Coef.	S.E.	T	*p*-value
Constant	0.3145	0.0299	10.524	0.000
Random effect	S.D.	Variance	*x* ^2^	*p*-value
Community-level	0.4634	0.2147	8934.4514	0.000
**Men model**				
Fixed effect	Coef.	S.E.	T	*p*-value
Constant	0.4963	0.0304	16.325	0.000
Random effect	S.D.	Variance	*x* ^2^	*p*-value
Community-level	0.4606	0.2122	4031.7811	0.000
**Women model**				
Fixed effect	Coef.	S.E.	T	*p*-value
Constant	0.1706	0.0309	5.514	0.000
Random effect	S.D.	Variance	*x* ^2^	*p*-value
Community-level	0.4791	0.2295	5380.8598	0.000

**Table 3:** Multi-Level Estimates: Trust Model.

Level	Variables	Pooled		Men		Women	
		Coef.	S.E.	Coef.	S.E.	Coef.	S.E.
	Constant	−0.295 ***	0.032	0.039	0.030	−0.242 ***	0.029
1	Trust	0.124 ***	0.014	0.091 ***	0.021	0.140 ***	0.016
	Trust*Population density	0.001	0.002	−0.000	0.003	0.002	0.002
	Trust*Land-use mix	−0.036	0.098	0.001	0.136	−0.083	0.117
	Trust*Area of parks	0.001	0.001	0.000	0.001	0.002 *	0.001
	Trust*Number of sports facilities	−0.001 **	0.001	−0.006 ***	0.001	0.003 ***	0.001
	Trust*% area for housing complexes	0.079	0.132	−0.125	0.182	0.232 *	0.136
	Age	0.013 ***	0.002	−0.024 ***	0.003	0.038 ***	0.003
	Age^2^	−0.000 ***	0.000	0.000 ***	0.000	−0.000 ***	0.000
	Gender (men)	0.202 ***	0.013				
	Education	0.015 ***	0.002	0.022 ***	0.003	0.009 ***	0.003
	Working status (working)	−0.038 **	0.017	−0.051 *	0.026	−0.039 **	0.019
	Length of residence	0.022 ***	0.005	0.006	0.006	0.032 ***	0.006
	Job (manual)	0.297 ***	0.023	0.207 ***	0.024	0.437 ***	0.029
	Household income	0.020 ***	0.005	0.026 ***	0.006	0.020 ***	0.006
	Number of household members	−0.050 ***	0.006	−0.056 ***	0.006	−0.040 ***	0.008
	Subjective health (healthy)	0.363 ***	0.015	0.425 ***	0.021	0.324 ***	0.019
2	Population density	−0.005	0.003	−0.004	0.004	−0.007 *	0.003
	Land-use mix	0.380 *	0.197	0.326	0.231	0.446 **	0.196
	Area of parks	−0.000	0.001	0.001	0.001	−0.001	0.001
	# of sports facilities	−0.002	0.001	0.000	0.001	−0.004 **	0.002
	% area for housing complexes	1.175 ***	0.306	1.130 ***	0.347	1.204 ***	0.301
	Fiscal self-reliance ratio	0.359	0.241	0.330	0.255	0.330	0.243
Variance Component	Level 2	0.18539		0.18656		0.19490	
	Level 1						

Notes: All level-1 variables were centered around group means, except for dummy variables. All level-2 variables were centered around grand means. This centering strategy was also applied to the reciprocity and network models. Due to the unique structure of a multi-level model, the relative magnitudes among independent variables’ coefficients cannot be compared by calculating standardized coefficients. *** *p* < 0.01, ** *p* < 0.05, * *p* < 0.1.

**Table 4:** Multi-Level Estimates: Reciprocity Model.

Level	Variables	Pooled		Men		Women	
		Coef.	S.E.	Coef.	S.E.	Coef.	S.E.
	Constant	−0.305 ***	0.031	0.009	0.030	−0.261 ***	0.029
1	Reciprocity	0.180 ***	0.015	0.147 ***	0.020	0.195 ***	0.018
	Reciprocity*Population density	0.003 **	0.002	0.006 **	0.002	0.001	0.002
	Reciprocity*Land-use mix	0.020	0.094	0.005	0.115	0.007	0.122
	Reciprocity*Area of parks	0.001 **	0.001	0.000	0.001	0.002 ***	0.001
	Reciprocity*Number of sports facilities	−0.005 ***	0.001	−0.006 ***	0.001	−0.004 ***	0.001
	Reciprocity*% Housing complexes	−0.280 **	0.118	−0.416 ***	0.156	−0.159	0.152
	Age	0.011 ***	0.002	−0.026 ***	0.003	0.036 ***	0.003
	Age^2^	−0.000 ***	0.000	0.000 ***	0.000	−0.000 ***	0.000
	Gender (men)	0.206 ***	0.013				
	Education	0.016 ***	0.002	0.022 ***	0.003	0.010 ***	0.003
	Working status (working)	−0.037 **	0.017	−0.047 *	0.025	−0.041 **	0.0019
	Length of residence	0.018 ***	0.005	0.002	0.006	0.029 ***	0.006
	Job (manual)	0.282 ***	0.023	0.192 ***	0.023	0.423 ***	0.029
	Household income	0.021 ***	0.005	0.025 ***	0.006	0.022 ***	0.006
	Number of household members	−0.050 ***	0.006	−0.055 ***	0.006	−0.040 ***	0.008
	Subjective health (healthy)	0.364 ***	0.015	0.426 ***	0.021	0.324 ***	0.018
2	Population density	−0.007 ***	0.002	−0.009 ***	0.001	−0.006 *	0.003
	Land-use mix	0.354 *	0.191	0.335	0.215	0.393 **	0.190
	Area of parks	−0.000	0.001	0.000	0.001	−0.001	0.001
	# of sports facilities	−0.000	0.001	−0.001	0.001	−0.000	0.002
	% area for housing complexes	1.489 ***	0.301	1.375 ***	0.336	1.565 ***	0.299
	Fiscal self-reliance ratio	0.422 *	0.245	0.381	0.257	0.412 *	0.248
Variance Component	Level 2	0.18629		0.18592		0.19661	
	Level 1						

*** *p* < 0.01, ** *p* < 0.05, * *p* < 0.1.

**Table 5:** Multi-Level Estimates: Network Model.

Level	Variables	Pooled		Men		Women	
		Coef.	S.E.	Coef.	S.E.	Coef.	S.E.
	Constant	−0.561 ***	0.033	−0.225 ***	0.032	−0.518 ***	0.033
1	Network	0.091 ***	0.004	0.092 ***	0.005	0.088 ***	0.005
	Network *Population density	−0.000	0.001	−0.000	0.001	−0.000	0.001
	Network *Land-use mix	−0.025	0.027	−0.047	0.033	−0.021	0.032
	Network *Area of parks	0.000	0.000	0.000	0.000	0.000 *	0.000
	Network *Number of sports facilities	−0.000	0.000	−0.001 **	0.000	0.000	0.000
	Network *% Area for housing complexes	−0.042	0.036	−0.078 *	0.045	−0.020	0.037
	Age	0.005 **	0.002	−0.030 ***	0.003	0.030 ***	0.003
	Age^2^	−0.000 ***	0.000	0.000 ***	0.000	−0.000 ***	0.000
	Gender (men)	0.246 ***	0.013				
	Education	0.017 ***	0.002	0.024 ***	0.003	0.011 ***	0.003
	Working status (working)	−0.004	0.017	−0.043 *	0.025	−0.003	0.018
	Length of residence	0.014 ***	0.005	−0.006	0.006	0.028 ***	0.005
	Job (manual)	0.258 ***	0.022	0.173 ***	0.022	0.401 ***	0.028
	Household income	0.023 ***	0.005	0.030 ***	0.006	0.023 ***	0.006
	Number of household members	−0.054 ***	0.006	−0.056 ***	0.006	−0.045 ***	0.007
	Subjective health (healthy)	0.357 ***	0.015	0.421 ***	0.021	0.319 ***	0.018
2	Population density	−0.004 *	0.002	−0.003	0.004	−0.005 **	0.002
	Land-use mix	0.477 **	0.208	0.511 **	0.226	0.501 **	0.221
	Area of parks	−0.000	0.001	0.000	0.001	−0.001	0.001
	# of sports facilities	−0.003 *	0.001	−0.002	0.002	−0.004 **	0.002
	% area for housing complexes	1.699 ***	0.332	1.695 ***	0.355	1.719 ***	0.336
	Fiscal self-reliance ratio	0.537 **	0.246	0.578 **	0.253	0.486 *	0.201
Variance Component	Level 2	0.18791		0.18471		0.19963	
	Level 1						

*** *p* < 0.01, ** *p* < 0.05, * *p* < 0.1.
